# Advancements in Engineering Planar Model Cell Membranes: Current Techniques, Applications, and Future Perspectives

**DOI:** 10.3390/nano14181489

**Published:** 2024-09-13

**Authors:** Sara Coronado, Johan Herrera, María Graciela Pino, Santiago Martín, Luz Ballesteros-Rueda, Pilar Cea

**Affiliations:** 1 Departamento de Química Física, Facultad de Ciencias, Universidad de Zaragoza, Pedro Cerbuna 12, 50009 Zaragoza, Spain; sara.coronado@correo.uis.edu.co (S.C.); johan.herrera1@correo.uis.edu.co (J.H.); 836152@unizar.es (M.G.P.); smartins@unizar.es (S.M.); 2 Centro de Investigaciones en Catálisis (CICAT), Escuela de Ingeniería Química, Universidad Industrial de Santander, Parque Tecnológico de Guatiguará, Km 2 vía El Refugio, Piedecuesta, Santander 681911, Colombia

**Keywords:** model cell membranes, vesicle fusion, Langmuir, Langmuir–Blodgett, proteins, enzymes

## Abstract

Cell membranes are crucial elements in living organisms, serving as protective barriers and providing structural support for cells. They regulate numerous exchange and communication processes between cells and their environment, including interactions with other cells, tissues, ions, xenobiotics, and drugs. However, the complexity and heterogeneity of cell membranes—comprising two asymmetric layers with varying compositions across different cell types and states (e.g., healthy vs. diseased)—along with the challenges of manipulating real cell membranes represent significant obstacles for in vivo studies. To address these challenges, researchers have developed various methodologies to create model cell membranes or membrane fragments, including mono- or bilayers organized in planar systems. These models facilitate fundamental studies on membrane component interactions as well as the interactions of membrane components with external agents, such as drugs, nanoparticles (NPs), or biomarkers. The applications of model cell membranes have extended beyond basic research, encompassing areas such as biosensing and nanoparticle camouflage to evade immune detection. In this review, we highlight advancements in the engineering of planar model cell membranes, focusing on the nanoarchitectonic tools used for their fabrication. We also discuss approaches for incorporating challenging materials, such as proteins and enzymes, into these models. Finally, we present our view on future perspectives in the field of planar model cell membranes.

## 1. Introduction

The cell membrane is far more than a mere physical barrier separating the interior and exterior of a cell. Biological membranes are dynamic and highly complex structures that play a crucial role in the survival, function, and cellular communication across all living organisms [1]. From the simplest bacteria to the most specialized cells of highly evolved multicellular organisms, the cell membrane is fundamental in a wide array of processes.

The cell membrane selectively regulates the passage of substances into the cell, maintaining an internal environment appropriate for cellular life and the proper execution of biological processes within the cell. In addition, it facilitates waste removal while preventing the ingress of harmful substances. Moreover, it plays an essential role in intercellular communication and signal transduction, enabling the coordination of activities among tissues and organs. It is also well recognized that the cell membrane plays a vital role in processes such as biosynthesis, detoxification, metabolism, signaling, sorting, cell–cell interactions, motility, pathogen defense, trafficking of lymphocytes, inflammatory response, and more. These functions arise from various physiological processes, driven by a combination of physical forces acting within the biomembrane, with the membrane composition playing a significant role in such a balance of forces [2].

For the aforementioned reasons, understanding the composition and function of the cell membrane is a primary goal of modern biology and medicine, together with the understanding of the physical chemistry phenomena of the surrounding aqueous solution and their relation with the membrane behavior and functions [3,4,5,6,7]. Such knowledge is crucial to grasp phenomena such as cellular communication and the intricate mechanisms of cell function. This comprehension can also facilitate the design of drugs that can be specifically recognized and internalized by target cells. However, working directly with cell membranes is often challenging and complex due to experimental difficulties, as well as other factors such as significant variations in composition between different cell types and between healthy and pathological cells [8].

In an attempt to gain a better understanding of the structural organization, mechanical properties, and functions of biomembranes, model cell membrane systems, including liposomes and vesicles, insoluble Langmuir monolayers, and supported lipid bilayers, have been extensively studied. These model cell membrane systems can greatly assist biomedical research, offering meticulous control over membrane composition. In addition, they enable the creation of artificial membranes with specific characteristics for the design of drug transport vehicles such as liposomes, laboratory-created extracellular vesicles, and more. However, modeling cell membranes is rather challenging due to several factors, including (i) the complexity of real membrane composition; (ii) the inclusion of lipid rafts (existence of ordered liquid crystalline lamellar phase micro or nanodomains among less-ordered lipid areas); (iii) the asymmetry of the membrane, having two leaflets with distinct phospholipid composition; and (iv) the inclusion of membrane proteins without disrupting their functionality due to the presence of solid supports [9,10,11,12,13,14,15,16,17,18].

Model cell membranes constitute a collection of systems designed to investigate and comprehend the operation of one of the most intricate and ubiquitous biological structures in living organisms: the cell membrane found in both eukaryotic and prokaryotic cells. Model cell membranes simulate the configuration and composition of these biological membranes and serve as tools to understand the interaction of various substances, such as drugs [19,20] or xenobiotics [21], with the membrane. Furthermore, laboratory-created membrane systems can be developed to mimic both healthy and diseased conditions, allowing for comparative studies of their functions and investigating how external agents or compounds might repair the damaged membrane. In addition, they can be utilized to engineer custom drug carriers enveloped by a model cell membrane, facilitating targeted internalization into specific cells [22].

**Figure 1:**
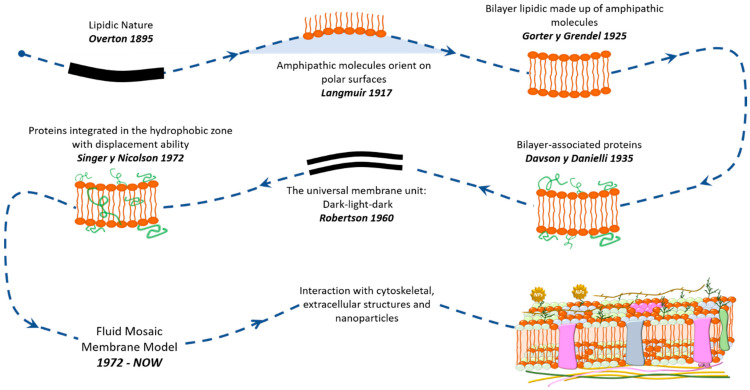
Timeline illustrating the development of various cell membrane models leading up to the currently accepted model. The last cartoon highlights current and future trends in the integration of extracellular structures into model cell membranes for diverse applications.

Since 1895, various models of cell membranes have been proposed. One of the earliest models was put forward by Overton, who suggested that the cell membrane functions as a lipid boundary with a hydrophilic nature [23]. This historical perspective reflects the understanding at that time, but it has since been refined and expanded upon with the development of more advanced models. The concept of the fluid mosaic model of cell membranes emerged in 1972 with the work performed by Singer and G. Nicolson [24]. Since then, the model considers the membrane as a fluid mosaic integrated by proteins with intra/extracellular domains in a lipid bilayer with a dynamic and malleable structure (Figure 1) [16,25,26]. This model has been developed through the discovery of new information, including membrane domains such as lipid rafts, protein aggregations, transmembrane glycoproteins, nanoclusters, viruses, cell junctions, and adhesion sites, as well as its cytoskeletal and extracellular interactions [16]. Further research is being conducted into the asymmetry, interaction, organizational schemes of mobility, and distribution of its natural components, in addition to external materials, such as nanoparticles (NPs) or drugs, due to their high potential in the field of medicine [9,10,11,12,13,14,16,18,27].

**Figure 2:**
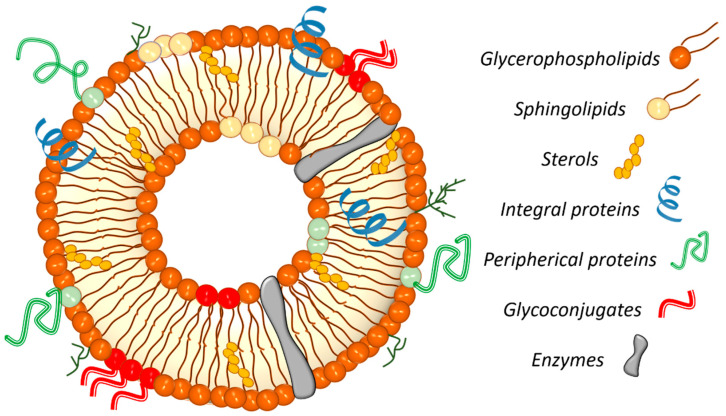
A simplified schematic diagram showing the composition of a cell membrane.

Cell membranes consist of a bilayer structure. Prokaryotic plasma membranes contain approximately 20–30% proteins and about 10% lipids [28]. In contrast, eukaryotic membranes have around 20% lipids by mass [29]. These amphiphilic molecules are crucial for providing structural support, flexibility, and fluidity to the membrane. The lipid composition includes glycerophospholipids (such as phosphatidylcholine, phosphatidylethanolamine, phosphatidylserine, phosphatidylinositol, and phosphatidic acid), sphingolipids (including ceramide, sphingomyelin, and glycosphingolipids), and sterols (cholesterol and stigmasterol) [1,28,30], as summarized in Table 1. These lipids act as a permeability barrier facilitating various biophysical and biochemical processes. The organization of lipid components in the membrane is heterogeneous, asymmetrical, and dynamic, influenced by their source (bacteria, virus, animal, or plant), functional roles, and sometimes nutritional deficiency or cellular apoptosis [30,31,32]. Importantly, eukaryotic cell membranes contain about 80% by mass of proteins [29], which perform specific functions such as molecular/ion exchange, enzymatic activity, communication, adhesion, reception, transmission, and signal transduction [30,33,34,35]. Proteins in the cell membrane are classified into integral and peripheral proteins based on their arrangement. Integral proteins partially or entirely traverse the cell membrane, while peripheral proteins are bound to lipid heads or integral proteins within the membrane [1,36,37]. Carbohydrates serve as recognition and binding points for other cells or external molecules, forming glycoconjugates through covalent attachment to the lipids and proteins within the bilayer [29,38,39]. Figure 2 illustrates a simplified schema of the composition of a cell membrane.

As highlighted above, membranes play a crucial role in living organisms, acting as both the protective layer and support for cells while also regulating numerous exchange and communication processes among cells and their external environment, including neighboring cells, tissues, and surrounding substances [1]. Given their involvement in processes of regulation, control, and communication, membranes have emerged as pivotal targets for research aimed at understanding such processes. Indeed, understanding and deciphering interactions among membrane components as well as these components and external compounds forms the cornerstone for the development of drug delivery systems [30]. However, due to the complexity and diverse array of compounds constituting real membranes, many investigations have directed their focus towards the development of model cell membranes that possess essential characteristics that ensure their validity and applicability in real systems, as discussed below. Spherical vesicles, or liposomes, delimited by a phospholipidic monolayer or bilayer separating two aqueous compartments, represent one of the simplest membrane models [40]. However, in this contribution, we aim to review planar model cell membranes, with special attention to the nanoarchitectonic tools employed for their construction and the specific insights each type of mimetic cell membrane can provide.

## 2. Classification of Planar Model Cell Membranes

Artificial systems for modeling of cell membranes have been extensively explored to assess and investigate the structure, composition, and functionality of this essential cellular barrier. The ultimate goal is to manipulate and control membrane systems for the development of therapeutic treatments for disease prevention, treatment, and monitoring [41].

Several platforms for mimicking cell membranes have been developed, with four main types of planar membrane models outlined in the scientific literature: lipid monolayers (LMs), supported lipid bilayers (SLBs), black lipid membranes (BLMs), and self-assembled monolayers (SAMs) [42]. Table 1 compiles the different types of planar model cell membranes, along with their respective pros and cons [6].

**Table 1:** Classification of planar model cell membranes into Langmuir monolayers (LMs), supported lipid bilayers (SLBs), black lipid membranes (BLMs), and self-assembled monolayers (SAMs).

Planar Model Cell Membranes	Advantages	Limitations	Cartoon	Ref
LM	Stable and facile to assemble, possessing a composition similar to real membranes.	LM just replicates one side of the bilayer, lacking the capability to functionalize transmembrane proteins.	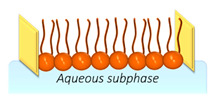	[43,44,45]
SLB	Easily to characterize and stable, capable of forming lipid domains, and amenable to functionalization with other substances.	Interference in interactions stems from the substrate effect, coupled with the inability to functionalize transmembrane proteins.	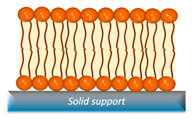	[46,47]
BLM	Free from substrate disturbances, allowing for functionalization by transmembrane proteins on both sides of the bilayer.	Prone to instability in the surrounding medium, leading to membrane fluctuations caused by variations in tension at the edges.	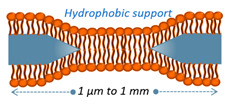	[48]
SAM	They readily incorporate cholesterol and experience minimal perturbation from the substrate due to the strong anchoring of molecules within the lipid layer to the underlying substrate.	High rate of lipid oxidation.	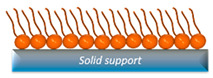	[46,49,50]

**Figure 3:**
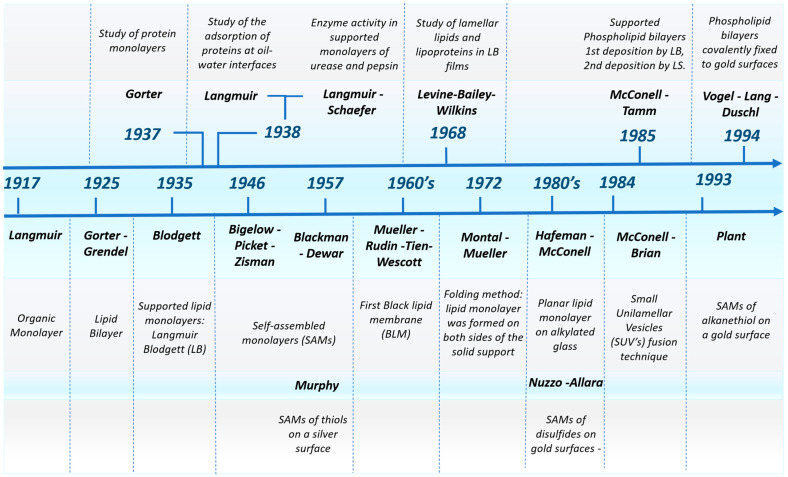
Timeline illustrating the most significant breakthroughs in the development of experimental planar model cell membranes.

In the following lines, we provide a brief historical overview of the key breakthroughs in the development of the most significant planar model cell membranes, with a timeline illustration of each experimental technique used in their fabrication (Figure 3). Later in the text, each of these methodologies will be explained in greater detail, along with recent advances in the field. Historically, the formal beginning of the scientific study and fabrication of monolayers was established with the pioneering work of Irving Langmuir in 1917 [51]. Langmuir discovered that oil molecules spread spontaneously across the water–air interface, forming a monomolecular layer. He was able to measure the thickness of this layer, providing the first empirical evidence of its monomolecular nature. Langmuir’s experiments also revealed that the hydrocarbon chains in the monolayers were not entirely flat on the water surface but rather curled, which was later a crucial insight into the flexible structure of lipid molecules. This work laid the groundwork for understanding the bilayer structure of cell membranes and established Langmuir films as a fundamental experimental model for studying biological membranes. The concept of the lipid bilayer was first developed in 1925 by Gorter and Grendel [52], who extracted lipids from red blood cells. Their experiments, which involved spreading these lipids on a Langmuir trough, revealed that the surface area of the lipid molecules was exactly half of what would be expected for a monolayer, leading to the conclusion that the cell membrane consists of two layers of lipids. This observation was instrumental in advancing the molecular understanding of cell membranes. In 1935, K. Blodgett [53] made another significant advance by transferring Langmuir monolayers from the air–water interface onto solid supports, creating what became known as Langmuir–Blodgett films. This technique allowed for the precise control and manipulation of monolayers on solid substrates, facilitating subsequent experimentation with membrane models. Two years later, Gorter conducted research into the characteristics of proteins and associated factors when these molecules are spread onto a water surface [54]. Langmuir made another significant contribution to the field in 1938 [55], studying the adsorption of proteins at oil–water interfaces and the preparation of protein–lipid membranes. Furthermore, in the same year, in collaboration with Vincent Shaeffer [56], he studied the enzymatic activity of urease and pepsin by immersing the respective enzyme-supported monolayers on coagulated milk curd and milk. In 1968, Levine and colleagues [57] employed the LB technique to prepare a dipalmitoyl lecithin bilayer. In this study, they observed the perpendicular organization of the lipid chains in the lamellar phase and confirmed the value of the LB technique for understanding the molecular interaction.

It was not till the 1960s when the first planar model cell membranes were fabricated, thanks to the pioneering work of Mueller et al. [58]. These authors prepared the first black lipid membranes that were formed at the aperture of a hydrophobic separator between two aqueous solutions, a technique that became foundational for studying membrane properties. The original method involved the “painting” technique, where a solution containing phospholipids, typically dissolved in a volatile solvent like decane, was applied across the aperture using a brush or syringe. The amphiphilic nature of the lipids caused them to self-assemble, with their hydrophilic heads facing the aqueous phases, forming a lipid bilayer at the interface. Decane was chosen for its high volatility and low viscosity, which facilitated partial evaporation and easier movement of the solvent away from between the lipid layers, ensuring proper membrane formation. However, this method often left residual solvent within the membrane, which could result in soft and highly elastic membranes. Efforts to reduce the residual solvent content included coating the aperture with amphiphobic agents, lowering the temperature to below the freezing point of the solvent, and using longer-chain solvents that were less likely to remain within the membrane. Additionally, creating asymmetric membranes where the two leaflets consisted of different lipid compositions required further refinement of the technique. In 1972, Montal and Mueller introduced a “folding” method to improve membrane formation [59]. In this approach, a lipid monolayer was first formed at the air–water interface on both sides of the membrane support using a lipid solution in a volatile solvent. The aqueous level was then raised, causing the two monolayers to meet at the aperture, thereby forming a bilayer structure. This method minimized the presence of residual solvents, producing membranes that more closely resembled natural biological membranes.

The 1980s brought further innovation in the field of planar model cell membranes with the development of planar model cell membranes by McConnell et al. [60]. McConnell’s work involved the transfer of a Langmuir–Blodgett monolayer onto solid substrates, followed by the formation of bilayers through additional deposition of a monolayer using the Langmuir–Schaefer procedure [61]. This methodology allowed for the creation of asymmetric bilayers, which more accurately represented the natural asymmetry found in biological membranes.

Another effective method for fabricating planar model cell membranes is the vesicle fusion technique. This approach, which was pioneered by McConnell and colleagues in 1984 [62], has become a fundamental technique in the study of supported lipid bilayers, providing a robust platform for investigating membrane-associated processes and protein interactions. Vesicles are first prepared and then dispersed into an aqueous solution that covers a hydrophilic substrate. Due to the favorable hydrophilic interactions, the vesicles spontaneously adsorb onto the substrate, which is followed by their rupture, spreading out to form a continuous planar lipid bilayer. This method can also be combined with the Langmuir-Blodgett technique to create asymmetric bilayers, where the lipid composition differs between the two leaflets.

On the other hand, the foundational work on self-assembled monolayers (SAMs) began with Bigelow in 1946 [63] and was further advanced by Blackman and Dewar in 1957 [64], who published key studies on the formation of these monolayers. Notably, a 1957 patent [65] described the assembly of thiols on a silver surface, marking a significant early achievement in this field. However, it was not until the 1980s that research on SAMs truly flourished, largely driven by the seminal contributions of Nuzzo and Allara [66]. Their work demonstrated the self-assembly of disulfides on gold surfaces, which, when combined with the advent of advanced microscopic characterization techniques, allowed for detailed analysis of these film structures. This breakthrough ignited widespread interest in SAMs and their potential applications. In 1993, Plant [67] fabricated a self-assembled alkanethiol monolayer on a gold surface, which served as a hydrophobic layer. Stable lipid bilayers were then formed on top of this layer through a vesicle fusion process, resulting in a hybrid system. This system offered several advantages, including easier preparation, greater reproducibility, long-term stability, and the capability to form on an electrically conductive substrate. Vogel and coworkers [68] prepared phospholipid bilayers that were covalently fixed to gold surfaces, which remained mechanically and chemically stable for several days to weeks. These results paved the way for the development of tethered bilayer lipid membranes (tBLMs) [69]. In these systems, a tethering molecule or anchor lipid covalently links the lipid bilayer to a solid support, effectively separating the bilayer from the substrate. This separation minimizes membrane–substrate interactions, providing a more physiologically relevant environment that allows for the functional incorporation of membrane proteins, thereby enhancing the utility of tBLMs in studying membrane dynamics and protein functions [70].

### 2.1. Lipid Monolayers (LMs)

Lipid monolayers at the air–liquid interphase represent one of the simplest platforms for modeling cell membranes. Typically created using nanoarchitectonic tools such as Langmuir methodologies (explained below in more detail), they simulate only one out of the two layers of the cell membrane, that is, either the outer or inner layer, but not both of them in a bilayer fashion. The key advantages of LM include: (i) easy incorporation of lipids, sterols, etc., which closely mimics the composition of a cell membrane [71]; (ii) they are ideal platforms for the analysis of intermolecular interactions between different membrane components within the membrane using thermodynamic, spectroscopy, and microscopy techniques [72]; (iii) they enable the demonstration and functioning of domains (also called lipid rafts) [73] and lipid reorganization [74]; (iv) permit the investigation of interactions between membrane components and species in the surrounding liquid media, such as ions [75], proteins [76], defense peptides [77], anesthetics [78], antifungals [79], xenobiotics [80], drugs [81], NPs [82], and more. The main limitations of these LMs are related to their inherent fragility, which affects both fundamental studies [83,84,85] and potential applications [86]. An effective response to this fragility is the construction of membranes on solid supports, which provide better mechanical stability, as described below.

### 2.2. Supported Lipid Bilayers (SLBs)

Supported lipid bilayers are regarded as attractive systems for mimicking cell membranes. This membrane model involves the adsorption of lipids onto a substrate, where they become properly oriented due to their amphiphilic nature, forming a stable bilayer. SLBs have been extensively used to investigate lipid interactions, functionalization with peptides, etc. [30,31]. They are typically formed using the vesicle fusion technique, leveraging the stability of bilayers formed by van der Waals or electrostatic interaction forces with the substrate [30,35]. However, it is essential to study the substrate compatibility beforehand, as bilayers may not form properly on common substrates such as gold [30] or titanium oxide [1]. Other methodologies employed for fabricating SLB include the LB technique, typically used for depositing the first layer, and the Langmuir–Schaefer (LS) technique, often employed for depositing the second layer [56]. Spontaneous conversion of a LB monolayer into a bilayer upon hydration has also been described as a suitable methodology for supported bilayer model cell membranes [87]. SLBs possess several key characteristics for their use as model cell membranes, including their ability to evaluate ligand–receptor interactions, their easy characterization through optical and electrochemical techniques [88], their great potential for coupling and studying sensory detection systems, as well as their potential for the investigation of phenomena associated with the coupling of antibodies with their respective antigens in combination with microfluidic systems [6]. Even though SLBs represent excellent platforms to study and model cell membranes, there remain gaps and challenges in mimicking and evaluating the functioning of certain components, such as transmembrane proteins. This is particularly evident due to the interference from substrate support, which limits their study. Such interference does not exist in the natural cell environment [89]. The use of porous supports can somehow circumvent this issue, as it allows for the fabrication of membranes that are suspended in the pores but supported by the non-porous regions of the substrate [90]. This approach may contribute to maintaining the functionality of incorporated proteins and provide the possibility of a liquid interface on both sides of the membrane. Examples of porous substrates include alumina, ultrafiltration glass, or polycarbonate membranes.

### 2.3. Black Lipid Membranes (BLMs)

Black lipid membranes consist of a lipid bilayer that fills a hole or aperture in a hydrophobic substrate (see cartoon in Table 1). Since the area of interest of the bilayer is located in a substrate-free region, this facilitates the localization of transmembrane proteins [91]. In addition, it enables the investigator to access either of the two faces of the membrane for functionalization. Despite these attractive characteristics, the formed bilayer is rather unstable due to limited control over membrane growth and may encounter challenges in characterization techniques that could potentially deteriorate the suspended bilayer. Although small fluctuations at the edges can induce membrane instability and even lead to rupturing, this model cell membrane enables the evaluation of ion interaction and exchange through electrostatic potential and conductance, as well as the effects of magnetic fields on lipid rearrangement [48]. Subsequent works in the field focused on eliminating the organic solvent used to fabricate and stabilize these suspended membranes [92,93,94,95,96], improving the stability and durability of these membranes by the fabrication of micro- and nano-tapered edge structures, as well as amphiphobic surface modification [97]. In addition, efforts have been made to increase the membrane area, facilitating the incorporation of target ion channels within the black lipid membrane [98]. Notably, the preparation of freestanding planar membranes on TEM (transmission electron microscope) grids has been recently reported. These planar model cell membranes offer improved stability and enable the simultaneous production of arrays of large-area membranes, allowing for rapid dynamic collection of data and statistical analysis [99,100,101].

### 2.4. Self-Assembled Monolayers (SAMs)

Self-assembled monolayers (SAMs) involve the spontaneous chemisorption of a lipid monolayer, typically functionalized with a terminal thiol group, onto a surface, often gold [102]. The substrate can also be pre-functionalized with other substances, such as N-heterocyclic carbenes, to enhance stability and evaluate molecule anchorage by reducing substrate interference [50,103]. In addition, substrates with anchoring phospholipids and a spacer molecule have been functionalized to facilitate subsequent protein anchoring on the monolayer surface, exemplified by DPhyTL (2,3-di-O-phytanyl-glycerol-1-tetraethyleneglycol-D, L-lipoic), which is an acid ester lipid [104]. The solvent exchange method relies on the formation of micelles in alcohol/water media through changes in solvent composition, such as the addition of water or removal of the organic component [105]. According to the literature, the solvent exchange method yields high-quality SAMs by controlling lipid growth through the modulation of solvent mixing rates [106]. Moreover, it also promotes cholesterol insertion into the systems, a critical aspect due to its high composition percentage in real cell membranes [107].

## 3. Techniques for the Preparation of Planar Model Cell Membranes

Multiple methodologies have been developed for constructing planar model cell membranes, including Langmuir, Langmuir–Blodgett, Langmuir–Schaefer, vesicle fusion, self-assembly, spin-coating, self-spreading, and combinations thereof [108,109]. However, the last two techniques present certain challenges in controlling the formation of a single lipid bilayer due to their operating principles. In the spin-coating method, small drops of a phospholipid solution are spread on the surface of a substrate, which is subsequently spun. The solvent evaporates, and a thin layer of phospholipid is formed, but precise control over the formation of a monolayer or an ordered bilayer is rather difficult [110]. In the self-spreading method, the bilayer is formed through hydrophilic interactions between the polar heads of the phospholipid and the substrate. In other words, phospholipids are coupled or self-organized onto the substrate at a liquid/solid interface due to the attractive forces of hydrophilicity. Although this methodology is easy to apply, it often results in the formation of multiple lipid bilayers due to the lack of control by the operator during the assembly process [111]. In the following sub-sections, the most convenient methods for the preparation of planar model cell membranes are described.

### 3.1. Vesicle Fusion Method

The vesicle fusion method is one of the most widely used techniques due to its simplicity, versatility, and accessibility. Moreover, it does not necessitate sophisticated equipment to produce high-quality lipid bilayers [42]. This methodology has found extensive application in the preparation of model cell membranes, particularly in analyzing the formation of lipid domains and membrane processes such as adsorption, self-assembly, and protein localization, as well as in understanding cell organization or immunological synapses [70].

**Figure 4:**
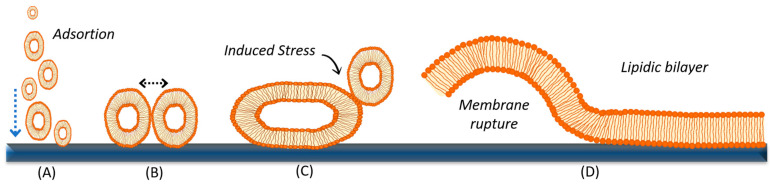
Mechanism of bilayer formation through the vesicle fusion method. (**A**) Vesicles are adhered and adsorbed onto the substrate. (**B**) Vesicles fusion. (**C**) Induced stress between neighboring vesicles. (**D**) Rupture of the vesicles to form a lipid bilayer onto the solid substrate.

The vesicle fusion method is based on the rupture and subsequent fusion of vesicles on the surface of a specific substrate such as quartz, mica, silica, gold, or titanium oxide, among others [112,113,114,115]. The fusion mechanism begins with the deposition of a vesicle solution onto the substrate surface, where they are adhered and adsorbed (Figure 4A). Subsequently, either spontaneously or under the influence of external factors, these vesicles undergo fusion, induced stress, rupture (Figure 4B,C), and possibly coalescence (Figure 4D), leading to the formation of bilayers. The adhesion and adsorption of vesicles are primarily determined by the strength of the attractive interactions between the vesicles and the support [116]. However, the stability of the adsorbed vesicles is influenced by the energy balance between the energy gained by adhering to the solid surface and the bending energy or rigidity of the vesicle membrane [116,117]. Upon deposition onto the support surface, several scenarios may occur, as illustrated in Figure 4:

(1) Vesicles may rupture if the mechanical stress induced by the support is sufficiently strong, leading to pore formation and subsequent nucleation until complete vesicle rupture [118].
(2) If the vesicles do not rupture and continue to adsorb, they may interact with each other and fuse, resulting in larger vesicles with a higher mean diameter until they reach a critical vesicular radius. At this point, the forces of bending and support attraction are strong enough to promote vesicle rupture (B) and the formation of discs or bilayer patches [116,119,120]. The fusion of vesicles with one another and their subsequent rupture is a complex process, and its occurrence depends on various factors, including the nature of the lipid components within the vesicles (lipid charge, polarity, headgroup size, acyl chain length, and degree of unsaturation); the size and concentration of the vesicles; the flow conditions; the nature of the substrate (hydrophilicity and roughness); osmotic stress; pH; and temperature [42]. Later in this section, we will discuss in more detail the different parameters that can be optimized in the laboratory to promote the formation of planar supported membranes by the vesicle fusion method.
(3) The bilayer patches formed are thermodynamically unstable due to their exposed edges, which can disrupt neighboring intact vesicles (C). This disruption promotes rupture and subsequent growth into a uniform lipid bilayer, a process known as coalescence (D) [119].

Considering the complexity of the rupture of vesicles and the mechanisms behind such a process, several experimental parameters play a crucial role in the formation of bilayers and need to be carefully controlled. As mentioned above, these parameters include temperature [121], pH [122], presence of ions [123], osmotic stress [124], nature of the support [125], lipid concentration [126], and vesicular size [127].

Temperature plays a critical role in the stability of vesicles as well as in the formation of lipid bilayers [121]. At temperatures above the transition temperature of the lipids (T_m_), the membrane enters a disordered fluid phase, which promotes vesicle–vesicle fusion [128]. In other words, due to the instability caused by the disordered fluid phase, vesicles reach their critical coverage more quickly, which promotes their deformation and the subsequent formation of bilayers [119,129,130]. In addition, if the temperature is reduced below the T_m_, a lipid bilayer with greater rigidity and stability is obtained. The transition temperature depends on the nature of the lipid mixture used in the preparation of the vesicles [30].

By regulating the pH of the solution in which the vesicles are located, the electrostatic repulsion forces between the lipid heads on the membrane can be modified, promoting attraction between the surface of the support and the vesicles, their deformation, and subsequent rupture, leading to the formation of bilayers on the surface of the substrate [131]. A similar phenomenon occurs with the ionic strength of the solution, which may be altered by a minimal concentration of divalent cations such as Mg^2+^, Ca^2+^, or Sr^2+^. These cations are located close to the hydrophilic head groups of the lipids in the membrane, increasing the electrostatic potential of the lipids and consequently promoting the attraction forces between the vesicles and a negatively charged solid substrate [42,119,123,132]. Another strategy to induce vesicle fusion is by generating an ionic force gradient through the vesicular membrane. This increases the membrane tension and promotes a difference in osmotic pressure, resulting in the rupture of the vesicles and consequently leading to their fusion and formation of bilayers [42,133].

The nature of the solid substrate on which the rupture of the vesicles occurs (roughness, hydrophilicity, surface charge, etc.) is another key parameter in the vesicle fusion and rupture processes. As reported by Richter et al., the formation of lipid bilayers is affected at the nanometric level by the roughness of the support. However, according to a study by Mornet et al., some bilayers ideally attach to the surface of a silica particle, even in areas where small roughness is present on a nanometric scale [112,134]. The physicochemical properties of the solid support, such as charge and hydrophilicity, promote the adhesion, mobility, and adsorption of the vesicles, key phenomena in the process of rupture and fusion. Bilayer patches formed on the silica surface exhibit certain sliding and mobility restrictions compared to the ease of sliding and displacement observed on mica supports. This phenomenon promotes additional stress at critical vesicular coverage, facilitating vesicular rupture [116,135]. In addition, some authors have reported that supports such as Au, SrTiO_2_, TiO_2_, and Pt hinder the process of rupture and fusion. In contrast, silicon-based supports (SiO_2_, Si_3_N_4_, and glass, among others) and mica are widely used in the vesicle rupture method as they facilitate adhesion, adsorption, and vesicular mobility [130,136]. However, it should be noted that some studies have successfully employed the vesicular fusion method on supports such as Au and TiO_2_ by carefully controlling external factors such as electrostatic force, surface functionalization, and the presence of potential differences due to electric fields [137,138]. Furthermore, new types of mechanically stable supports have been developed to reduce adsorption limitations, including porous materials, polymers, aerogels, xerogels, colloid crystals, and others [139].

The vesicle fusion method offers several advantages, including simplicity, versatility, and accessibility, leading to the reliable production of high-quality supported bilayers with minimal equipment. However, this technique has certain limitations regarding the nature of the lipid components forming the vesicles. Complex mixtures of lipids may result in vesicles with more defects [140], affecting the organization and anchoring of proteins or peptides [141]. In addition, these lipid mixtures can influence other physical properties, such as membrane curvature, which is determined by the ratio between the headgroup and the hydrophobic acyl chain of the lipids. This ratio dictates whether the lipids have a conical, cylindrical, or conical-inverted shape, subsequently affecting the curvature of the membrane formed from them, which can be planar, concave, or convex [142].

### 3.2. Langmuir Technique

**Figure 5:**
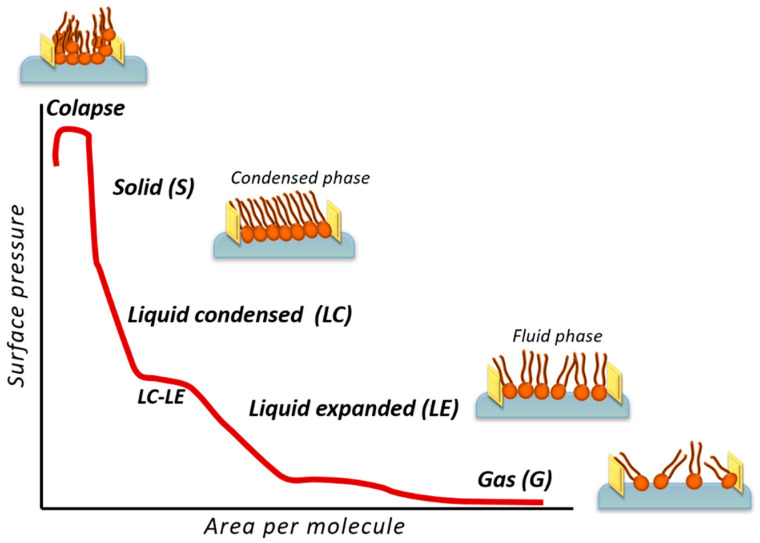
Illustrative plot of a Langmuir isotherm showing various phases and phase transitions typically observed in a surface pressure vs. area per molecule isotherm.

The Langmuir technique allows the preparation of highly ordered monolayers of amphiphilic materials at the air–water interface. This technique permits the fabrication of monolayers incorporating phospholipids, sterols, and other materials naturally found in cell membranes, such as proteins, peptides, and polysaccharides [143]. The instrument used to prepare these floating monolayers is called a Langmuir trough. To prepare a monolayer at the air–liquid interphase, or a Langmuir film, a solution containing the desired components of the model cell membrane in a volatile solvent is spread onto the water surface. After waiting a few minutes for the solvent to evaporate, slow compression of the film begins by moving the barrier(s) of the Langmuir trough. Upon the compression process, the surface pressure, π, is recorded versus the area per molecule, *A*, to obtain the so-called compression isotherm. The surface pressure is defined as the difference between the surface tension of the pure water and the surface tension of the water with the film. Figure 5 shows a representative π–*A* isotherm, although the appearance of all the phases and phase transitions shown in this figure depends on the specific material and conditions (such as the temperature, the nature of the subphase, and the nature of the surfactants). At surface pressure values close to zero, lipids exhibit poor cohesion, with a surface having domains of molecules and many uncovered areas, which is referred to as a gas phase (G). At higher surface pressures, the surface density increases and the molecules are more ordered and packed, moving from a liquid-expanded (LE) to a liquid-condensed (LC) and eventually to a solid phase (S) [144], as illustrated in Figure 5.

**Figure 6:**
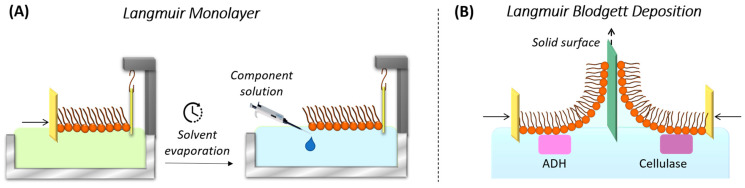
(**A**) Injection of proteins or enzymes beneath the monolayer to promote their incorporation into the planar model cell membrane. (**B**) Transfer of the monolayer, incorporating proteins or enzymes, onto a solid support. Adapted with permission from [164]. Copyright 2014 American Chemical Society.

In addition to the π–*A* isotherm, the formation of the monolayer can be studied in situ using other methodologies, including surface potential isotherms, UV-vis or FTIR reflection spectroscopies, Brewster Angle Microscopy (BAM), X-ray, and neutron scattering measurements. Importantly, theoretical models (e.g., molecular dynamics simulation) are excellent complements to experimental data, helping to construct models of the molecular organization in the membrane and to explain experimental observations. As described in more detail below, thermodynamic studies of mixed films at the air–water interface provide relevant information about the interactions between components, lipid rafts, complex formation, and the stability of the mixed films. The inclusion of different components in the model cell membrane can be performed using several strategies, including (but not limited to, as we will discuss later), the co-dispersion of the components to form a mixed Langmuir film, and the injection of proteins or other elements that are subsequently adsorbed at the interface (Figure 6A).

The co-dispersion of two or more components on the water surface results in mixed monolayers whose behavior can be compared to that of the pure monolayers to determine thermodynamic excess properties (excess areas, excess Gibbs energy of mixing, and excess Helmholtz energy of mixing), which provide relevant information about molecular interactions between these components [145,146].

Specific studies reported in the literature, with some illustrative examples gathered in Table 2, make use of Langmuir monolayers to evaluate the therapeutic potential of new substances and investigate potential adverse effects of anesthetics [147,148], impulse blocks [149], and raft formation [150,151]. Furthermore, thermodynamic studies exploring the interactions between membrane components and between the model cell membrane and drugs or xenobiotics have been conducted, including calculations of free energy changes, lateral diffusion coefficients, elasticity, and membrane compression [71,74].

**Table 2:** Illustrative examples of studies reporting interactions between planar model cell membranes (Langmuir monolayers) and various therapeutic agents.

Systems	Aim of the Work	Reference
Anesthetics	Evaluation of the interaction of lidocaine with a lipid monolayer composed of POPC and cholesterol in a solvent mixture to demonstrate its effect on packaging and permeability.	[78]
Phospholipidic drugs	Study of the action of HePC, a phospholipid used as a treatment against *visceral leishmaniosis,* on POPC monolayers and sterols to evaluate HePC affinity for the parasite membrane.	[152]
Antifungals	Recreation of a fungal membrane using POPC and sterols to evaluate the interaction of AmB and its effect on the formation of lipid rafts and pores through which ions pass, triggering cell death.	[79]
Antifungal Antibiotics	Study of AmB and Am3 interactions with lipids and cholesterol/ergosterol into the model cell membrane for understanding its biological activity and mechanism of action.	[153,154,155]
Lipid mixtures	Reconstruction of the microbial membrane of *E. coli* using a monolayer formed with varying percentages of PE, PG, and CL to study their interactions and thermodynamic properties.	[74]
Antineoplastic drug	Study of the interaction of paclitaxel in monolayers formed by ternary mixtures of DPPC, cholesterol, and sphingomyelin and its effect on compressibility and lipid raft formation as a function of cholesterol concentration.	[156]
	Study of the interactions of docetaxel in DPPC monolayers at several surface pressures to evaluate its absorption and penetration ability into the phospholipid matrix.	[20]
Antiprotozoals	Use of PTF as a treatment agent for Chagas disease by reconstructing a protozoal monolayer from DPPG to study its cytotoxicity and its effects on lipid fluidity and rearrangement.	[157]
Antiparasitic	Cyclosporine A, an immunosuppressive agent that has been studied to analyze its potential to be incorporated into model cell membranes that inhibit the development of the parasite.	[19,158,159]
Monoterpenoids	The incorporation of thymol (a biocidal drug) in monolayers formed by DPPC with analysis of the effect on the physicochemical properties of the membrane.	[160]
Antimicrobial peptides	Analysis of the interaction of defense peptides that target the cell membrane and organelles of malignant cells, altering their metabolism.	[45,77]
Anti-inflamatory drugs	Study of the interaction of ibuprofen with a phospholipidic monolayer (DPPC and DPPG) probing that ibuprofen penetrates into the hydrophobic region of the monolayer, accompanied by a fluidizing effect.	[161,162]
Anesthetics	Studies at the air–water interface of lidocaine with model cell membranes incorporating DPPC, DPPE, and SM indicate that the most probable mechanism of anesthetic action is the adsorption of lidocaine to the protein ion channel of the membrane.	[148]
Anti-histaminic drugs	Olopatadine and ketotifen interactions with the components of a model cell membrane offer information for the mechanism of action of these compounds.	[163]
Xenobiotics	Analysis of the interactions of curcumin with a model cell membrane (DPPC+Chol) probing that this compound tends to fluidize the monolayer.	[21]

POPC: 1-Palmitoyl-2-oleoylphosphatidylcholine; HePC: hexadecylphosphocholine; PE: phosphatidylethanolamine; PG: phosphatidylglycerol; CL: cardiolipin; DPPC: dipalmitoylphosphatidylcholine; DPPS: dipalmitoylphosphatidylserine; PTF: poly-thymolformaldehyde; DPPG: dipalmitoylphosphatidylglycerol; AmB: amphotericin B; Am3: amphodinol3.

### 3.3. Langmuir–Blodgett (LB) and Langmuir–Schaefer (LS) Technique

LB and LS deposition methodologies are relevant methods for generating mono- and bilayers immobilized onto a solid support, minimizing the number of defects. Importantly, leaflet asymmetry can be introduced by combining LB for the first layer and LS for the second one [56,61,165,166].

**Figure 7:**
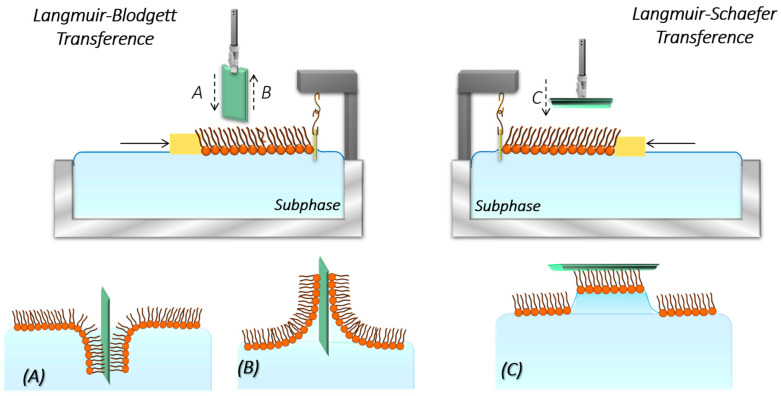
(Left, (**A**,**B**)) Langmuir–Blodgett and (right, (**C**)) Langmuir–Schaefer methodologies.

In the LB method, the deposition process occurs by withdrawing or immersing the solid substrate perpendicularly to the water subphase of a Langmuir trough once the target surface pressure for the monolayer at the air–water interphase is reached (Figure 7A,B) [53]. For hydrophilic substrates, the substrate is typically withdrawn from the water subphase, causing the polar heads of the film components to be deposited onto the substrate surface. Ideally, a second layer should be deposited if the substrate is reintroduced into the trough subphase. However, with phospholipids, a peel-off phenomenon often occurs during the deposition of the second layer, resulting in poor deposition and disorder, which can disrupt the first layer. In contrast, the second layer can be effectively deposited using the LS methodology [61,167], where the substrate is brought into contact with the monolayer in a parallel manner, Figure 7C. For instance, Kriechbaumer et al. used the Langmuir–Schaefer technique to deposit the system formed by neuronal cells, cancer cells, and chloroplasts with their respective ligands or chaperones on Cr/Au-coated glass slides for quantifying molecular interactions using total internal reflection ellipsometry (TIRE) without the need for labeling, protein purification, or reconstitution of membrane proteins [168].The combined LB and LS methodologies facilitate the formation of well-ordered bilayers that also exhibit the leaflet asymmetry observed in real cell membranes [169]. Interestingly, Caseli, Goto, and Rodrigues et al. used the Langmuir–Blodgett method to immobilize enzymes before depositing the lipid monolayer onto the solid support by injecting the enzyme solution just below the water–lipid monolayer interface [164,170,171], as illustrated in Figure 6B.

Although the formation of bilayers via LB/LS yields high-quality artificial membranes with minimal surface defects, several parameters and characteristics of the equipment and substances involved must be carefully considered as they significantly influence the final model cell membrane. In the case of phospholipids and substrates, their selection and treatment, respectively, are crucial to promote the formation of stable bilayers: lipids should have moderately long hydrophobic chains to prevent dissolution in the medium, yet not too long to prevent crystallization. Ideally, these lipids should be in gel phase rather than in a liquid or transient phase to ensure a proper coupling. Regarding the substrate, typically hydrophilic, it should possess a soft, low-roughness, flawless surface that is meticulously clean [165].

Regarding operational parameters in the LB and LS techniques, four of them are of high importance: surface pressure, area per molecule, contact angle, and transfer ratio. Proper control and monitoring of these parameters are crucial for the preparation of defect-free mono or bilayers. Surface pressure quantifies the interactions of molecules within the monolayer and between the monolayer and the underlying subphase. The contact angle refers to the angle formed between the liquid in the Langmuir trough and the surface of the substrate. According to the literature, it is preferably between 95° and 110° for immersions and between 50° and 60° for emersions [172]. The transfer ratio is a critical parameter for monitoring the deposition of the monolayer at the air–water interphase when transferred to the substrate. Ideally, its value should be close to 1, indicating that the area occupied by the transferred monolayer on the substrate matches the area occupied at the air–water interphase [144]. Higher values of the transfer ratio indicate monolayer instability at the air–water interface or collapse of the monolayer upon perturbation by the substrate. Lower values suggest poor transfer of the Langmuir monolayer onto the solid substrate.

## 4. Incorporation of Membrane Components into Planar Model Cell Membranes

### 4.1. Membrane Components and Their Role

Considering the diversity and heterogeneity of molecules constituting cell membranes, their composition varies depending on the type of membrane (eukaryotic, prokaryotic, organelles, etc.) and even between healthy and diseased cells [30,31]. For these reasons, simulating membrane structures presents significant challenges. However, by implementing various methods to prepare cellular models that mimic basic configurations, such as lipid bilayer/monolayers or supported membranes, it becomes possible to approximate and study the behavior of models that mimic real cell membranes. Over time, diverse methodologies have been developed for film preparation tailored to mimic the basic structure of cell membranes composed of binary or ternary lipid systems. These methods have evolved with modifications that incorporate factors such as surfactant concentration, divalent cations (Mg^2+^ and Ca^2+^), AH peptide, fluidic microchannels, and variation in osmotic pressure [42,173,174,175,176]. This flexibility has expanded the range of components interacting with lipid films that constitute cell membranes. During the last two decades, various membrane component incorporations have been reported, including glycerophospholipids, sphingolipids, proteins, enzymes, sterols, and ceramides (Table 3).

**Table 3:** Representative examples of various membrane components integrated into model cell membranes. A color code has been assigned to each family of components for better visualization. This code is maintained in Table 4, Table 5 and Table 6 to enhance the correlation between the information provided in both tables.

Glycerophospholipids		Sterols	Sphingolipids	Enzymes	Proteins/Glycoproteins
1,2-Dimyristoyl-sn-glycero-3-phosphocholine (DMPC) [137,177]	1-palmitoyl-2-oleoyl-sn-glycero-3-phosphocholine (POPC) [27,178,179,180,181,182]	Cholesterol (Chol) [27,126,149,150,153,155,178,183]	Glycosphingolipids: Ganglioside GM1 [179]	Alcohol Dehydrogenase (ADH) E. coli [164]	Enterotoxin: Cholera toxin b-subunit (CTB) [184]
1,2-dipalmitoyl-sn-glycero-3-phosphocholine (DPPC) [183,185,186]	1-palmitoyl-2-oleoyl-sn-glycero-3-phosphoethanolamine (POPE) [42]	7-ketocholesterol (7-KC) [178,187]	Ceramide galactosylceramide (GalCer) [179]	Cellulase [164]	Ephrin-A5 Fc Chimera (CF) [181]
1,2-dioleyl-snglycro-3-phosphocholine (DOPC) [173,182,188]	1-palmitoyl-2-oleoyl-sn-glycero-3-phospho-L-serine (POPS) [42]	Ergosterol [154,155]	Brain sphingomyelin (BSM) [42]	Catalase [170]	Annexin A5 (AnxA5) [173]
1,2-distearoyl-sn-glycero-3-phosphatidylcholine (DSPC) [189,190]	1,2-dioleoyl-sn-glycero-3-ethylphosphocholine (DOEPC) [173]	25-hydroxycholesterol (25-OH) [187]	Sphingomyelin (SM) [27,158,182,187]	Tyrosinase [191,192]	gp41-antibodies 2F5/4E10 MPER peptide [182,193]
1,2-diooleoyl-sn-glycero-3-phosphoserine (DOPS) [173,194]	1,2 ditetradecanoyl-sn-glycero-3-phosphate (DMPA) [177]	7β-hydroxycholesterol (7β-OH) [187]		Urease [171]	Type I collagen (rat tail) [195]
1,2-diooleoyl-sn-glycero-3-phosphatidylglycerol (DOPG) [173]	1,5-Odihexadecyl-N-succinyl-L-glutamate (DHSG) [196]			Horseradich peroxidase [197,198,199]	Glycosylphosphatidylinositol (GPI) anchored [200]
1,2-dilauroyl-sn-glycero-3-phosphocholine (DLPC) [179]	Dipalmitoyl phosphatidylserine (DPPS) [5]			Asparaginase [201]	Heparan sulfate proteoglycan HSPG [202]
L-α-Phosphatidylethanolamine (PE) [203,204]	1,2-dipalmitoyl-sn-glycero-3-ethylphosphocholine (DPEPC) [194]				β-lactoglobulin [205]
Phosphatidylglycerol (PG) [204]	Cardiolipin (CL) [149,204,206]				α-lactalbumin (α-LA) [207]
Dipalmitoyl phosphatidylglycerol (DPPG) [170,171,197,198,208]	1,2-Dipalmitoyl-sn-glycero-3-phosphoethanolamine-N-(glutaryl) (DP-NGPE) [195]				Syndecan-4 [202]
L -α–phosphatidylinositol (PI) [206]					G-protein-coupled receptors [168]

**Table 4:** Illustrative examples of monolayer-type model cell membranes categorized with indication of membrane components, fabrication method, and research objectives (the color code for the membrane components is aligned with Table 3).

Membrane Components	Study objective	Ref.
Langmuir Films		
DPPE:GM1-CTB	Membrane study: Evaluate DPPE:GM1 lipid monolayers before and during the binding of cholera toxin (CTB5) by neutron reflectivity.	[184]
PE:PG (3:1) and CL (5–20%)	Membrane study: Effect of another component on the organization and properties of the cell membrane of large negative bacteria.	[204]
7-KC, Chol, SM, and POPC	Membrane study: Role of oxysterols in neurodegeneration. Incorporation of oxysterols changes membrane permeability and fluidity, disrupting synaptic transmission in cells and leading to neuronal dysfunction.	[178]
DPPC and Syndecan-4 HSPG	Membrane study: Role of syndecan-4 in simplified models of cell membranes in order to access molecular interactions. Interaction of heparan sulfate proteoglycan involved in biochemical processes at the level of cell membrane surfaces.	[202]
POPC, SM, Chol, and trans-resveratrol	Drug delivery: Effects of resveratrol in physicochemical parameters of model membranes at different concentrations on model membranes. Analysis of resveratrol penetration and interaction with the lipid monolayer.	[27]
DPPC, DPPG, and Chol Mehylene blue MB and Acridine orange AO	Treatment of diseases and encapsulation: MB and AO incorporation into liposomes for photodynamic therapy against cancer. Analysis of molecular-level interactions between MB or AO and lipid monolayer using surface pressure isotherms and PM-IRRAS.	[208]
PE and penicillin	Drug delivery: Evaluation of the interaction of nano-penicillin G spheres with a lipid monolayer composed of PE from E. coli to demonstrate its effect on penetration and membrane fluidity.	[203]

**Table 5:** Illustrative examples of supported monolayer-type model cell membranes categorized with indication of membrane components, fabrication method, and research objectives (the color code for the membrane components is aligned with Table 3).

Membrane Components	Molecular Incorporation Method	Method—Support Type	Study Objective	Ref.
Supported Monolayer				
Palmitic acid-PA, normal human lung cells MRC-5, 2-methyltriclisine (drug)	Langmuir monolayer PA	Langmuir–Blodgett MRC-5-Mica	Drug delivery: Study of effects of bioactive compounds over cellular cultures. Analysis of palmitic acid monolayer stability after drug incorporation. Effect of the presence of drug in LB layers on the development of human normal lung fibroblast cells.	[209]
DPPG, HRP, and chitosan	Langmuir monolayer: The enzyme solution was injected in the subphase under a pre-formed lipid monolayer	Langmuir–Blodgett Optical glass Gold AT-cut quartz crystal coated with Au	Biosensor: Study of HRP immobilization in DPPG and chitosan monolayer to evaluate activity preservation and biosensing by Langmuir monolayers and LB films.	[197,198]
DMPA and DMPC	Preparation of vesicles: Lipid hydration	LB	Study of DMPC liposomes interaction with a DMPA monolayer by LB technique.	[177,210]
DPPC, DPPA, DPEPC, DOPS, 1,2-dihexadecanoyl-3-trimethylammonium-propane (DPTAP)/ pluronic F-127 cubosomes	Langmuir monolayer	LB	Drug delivery: nanoparticles: Analysis of molecular interactions between different model membranes (DPPC monolayer, DPPA monolayer, etc.) and cubosomes (LLCNPs) as alternative, biocompatible drug delivery systems by Langmuir technique.	[194]
DPPC, DPPS, PI, CL, SM, TAT–ritonavir-loaded poly (L-lactide) NPs	Langmuir monolayer	Injection NPs: Langmuir–Schaeffer—Silicon substrate	Drug delivery: Analysis of biophysical interactions of NPs with the model cell membrane to determinate their ability in cellular delivery of the encapsulated therapeutic agent (ritonavir).	[206]
Cis-9-octadecenoic acid (OA), α-LA, CaCl_2_ (Ca^2+^)	Langmuir monolayer CaCl_2_ was dissolved into suphase before spreading of amphiphilic molecules	LB-solid support	Drug test: Study of monolayer interactions with proteins and metal ions that affect the stability of Langmuir monolayers and the LB film and fabricate well-defined structures. Effects of pH, temperature, and the density of molecular packing on the ability of fatty acid and protein to form an antitumor complex at the interface.	[207]
DPPC and β-sheet peptide nanofibers NFs	Langmuir monolayer	Langmuir: Suspension NFs was injected slowly into the buffer subphase	Drug delivery: nanoparticles: Effect of the interactions between three types of NFs with different ethylene glycol lengths and DPPC monolayers, due to the interactions determine the cellular association and toxicity of the NFs.	[211]
DPPC and hydrophobic fumed silica NPs	Langmuir monolayer	Langmuir: Spreading of SiO_2_ NPs	Nanoparticles: Evaluation of the effect of hydrophobic fumed silica nanoparticles on the thermodynamic, structure, and rheological properties of DPPC monolayers.	[186]
DPPC and chitosan, PVA, functionalized Fe_3_O_4_ NPs	Langmuir monolayer	Langmuir: Spreading of NPs	Nanoparticles: Effect of NPs interactions with the model cell membrane. Analysis of polymer biocompatibility in NPs according to its adsorption process into the model cell membrane.	[185]
(GPCRs: CXCR4) -Ishiwaka cells/BSA—CXCL12 α	Double incubation, first the cells and then the ligand CXCL12α	LS-Cr/Au-coated glass slides	Drug delivery: Quantify interactions using TIRE of receptors within native cell membranes and ligand or drug interactions targeting GPCRs, which are targeted by approximately 60% of all therapeutic drugs.	[168]
DPPG–Ureasa	Langmuir monolayer based on the injection of molecular solutions below the air–water interface after having spread the lipid components and evaporated the solvent.	LB–quartz crystal, quartz plate, indium tin oxide (ITO) substrates	Biosensor: Evaluation of urease enzymatic activity via urea molecular recognition ability. Colorimetric assays to demonstrate the sensing capability of the films DPPG-ureasa, according to the results after 1 week, the urease–DPPG activity was preserved in 93%.	[171]
DPPG–Catalase		LB-optical glass and gold	Biosensor: Evaluation of DPPG-Catalase enzymatic activity to degrade H2O2 via redox reaction to prevent its accumulation and cellular damage associated with neurodegenerative diseases, cancer, or diabetes.	[170]
ADH/cellulase− DPPC		LB-solid glass supports	Membrane study: Enzymes in the production, identification, and/or control of second-generation ethanol. After the PM-IRRAS analyses, the conformations of these enzymes into lipid LB films are affected in the presence of cellulose and ethanol. After 20 days, ADH/cellulase activity in DPPC−cellulase−ADH SLB was preserved in 85% vs. 45–60% in homogeneous solution.	[164]

**Table 6:** Illustrative examples of supported bilayer-type model cell membranes categorized with indication of membrane components, fabrication method, and research objectives (the code color of the membrane components is aligned with Table 3).

Membrane Components	Molecular Incorporation Method	Method—Support Type	Study Objective	Ref.
Lipid Bilayer				
Sulfated butyl oleate (SBO), phospholipids, and β-lactoglobulin	Electrostatic SA: First layer: SBO Second layer: SBO, or phospholipids	-	Vehicle for bioactive substances (nutritional, pharmaceutical, and/or cosmetic applications): The patent relates the structures obtained from protein and emulsifier interaction, more particularly to structures comprising a protein supramolecular core coated with at least a lipid layer. The invention also encompasses methods for obtaining these structures and food compositions comprising them.	[205]
DOPC, DPPC, ciprofloxacin, and moxifloxacin	SUV preparation: lipidic hydration	Vesicle fusion and rupture method-mica	Drug test: Evaluate the interactions between fluoroquinolones with the DOPC/DPPC bilipid layer by studying the drugs ability to diffuse through membranes. Analysis of changes in membrane properties based on the conformation and orientation of lipid chains with the drugs.	[212,213]
POPC, Ephrin-A5 Fc Chimera (CF)	SUV’s fusion and rupture method (POPC): Clean and O2-plasma activated glass cover slips	Detergent-mediated reconstitution method: NOG-EA5/Fc proteoliposomes	Membrane study: *Development of a biomimetic platform* that enables culturing primary neurons and testing cell surface-receptor ligand interactions. The POPC-EA5/Fc SLB initiates adhesion and facilitates neuronal growth.	[181]
POPC or DOPC: SM: Chol Interaction with gp41-2F5/4E10	SUV’s fusion and rupture method-mica	Addition and incubation on SLB	Vaccine development *to HIV-1 inhibition*: the mAbs show the ability to be intrusive and induce confined local disorder in the membranes.	[182]
DPPC/POPC and GM1- CTB	Vesicle fusion and rupture method-SiO_2_/Si substrate		Evaluation technique for the characterization SLB: Study of CTB-GM1 and POPC/DPPC interactions using Ellipsometry.	[179,214]
DOPC, DOPS, DOPG, DOEPC, and annexin A5 (AnxA5)	Vesicle fusion and rupture method (addition of divalent cations and osmotic gradients)-SiO_2_	Injection and adsorption on SLB	Evaluation technique for the characterization of SLBs: Analysis and monitoring of molecular interactions by Quartz Crystal Microbalance QCM.	[173]
DP-NGPE: POPC and type I collagen (rat tail)	SUV’s fusion and rupture method-SiO_2_		Membrane study: Development of a biomimetic platform to study the interactions between extracellular matrix components and cells. Conjugated type I collagen maintains the growth and adhesion of smooth muscle cells on the lipid bilayer platform.	[195]
DLPC- GalCer	Vesicle fusion and rupture method: SiO_2_/Si substrate		Evaluation of DLPC-GalCer coexistence phases using Ellipsometry. Organization of clusters GalCer in DLPC bilayer.	[179]
POPC, POPE, POPS, BSM, Chol	Use of AH peptides for vesicle fusion: Silica substrate		Simple technique for preparation for SLB, as model cell membrane of HIV-1.	[42]

#### 4.1.1. Lipids

Since lipids are responsible for the structure and support of the cell membrane, and considering that more than 1000 different types of lipids are reported in eukaryotic cells [30,215,216], it is essential to increase the number of lipid components included to simulate the structure of a real cell membrane more accurately. In this context, Hardy et al. [42], motivated by the potential use in the design of antiviral vaccines, successfully modeled the cell membrane of the HIV-1 virus as a supported bilayer composed of five lipid components: POPC, POPE, POPS, BSM, and Chol. To this end, these authors implemented a vesicular fusion method induced by the AH peptide to determine the effect of another component on the organization and properties of the cell membrane of Gram-negative bacteria [42]. Wydro et al. prepared monolayers composed of a ternary lipid system formed by POPE:POPG or DPPG (3:1) and CL (5–20%). Their study showed that high concentrations of CL weaken the molecular interactions to a greater extent between POPE:POPG than between POPE:DPPG [204].

Since the 1970s, research into the interactions between sphingolipids, sterols, and proteins has been crucial in understanding the organization principle of cell membranes [151]. In 1997, Simons and Ikonen [73] proposed a new structural model of the cell membrane, which is based on the clustering of cholesterol and sphingolipids to form rafts that are selectively attached to proteins that move within the fluid bilayer. These lipid rafts are specialized platforms that facilitate selective membrane transport and the spatial and temporal regulation of signaling pathways [17,73,151]. Signaling pathways are responsible for the patterning of the entire organism, including embryonic and brain development, as well as neurological disorders and cancer [17,217,218]. In 2017, the effect of cholesterol oxidation in neurodegenerative diseases was studied by incorporating 7-KC, a product of Chol oxidation, into a monolayer composed of the most common lipids in neuronal membranes (SM and POPC). This study found that incorporation of 7-KC increases the intermolecular interactions with lipids, affecting the physicochemical properties of the membrane and contributing to neuronal dysfunction [178]. Then, in 2022, Wnętrzak et al. [187] discussed the effects of three oxysterols, 7-KC, 7β-hydroxycholesterol (7β-OH), and 25-hydroxycholesterol (25-OH), in the stabilization of SM:Chol lipid rafts using the Langmuir monolayer technique. Their findings revealed that 7β-OH and 7-K caused a significant raft destabilization, leading to cell death. In contrast, 25-OH demonstrated a stabilizing effect on the raft due to its low toxicity compared to the other oxysterols.

#### 4.1.2. Proteins

Since the interaction between proteins and the lipid bilayer is one of the most relevant aspects of cell membrane functioning, several authors have performed studies to analyze intermolecular interactions with different proteins for clinical applications. One example is the interaction between HIV-1 virus 2F5/4E10 antibodies with the peptides of the proximal membrane of the gp4 protein and the lipid bilayer formed by DOPC:SM:Chol to evaluate the use of peptides as antigens in vaccines for the treatment of the virus [182]. With the purpose of promoting cell growth, in 2010, a biomimetic platform composed of a DP-NGPE:POPC lipid bilayer functionalized with type 1 collagen was implemented for the adhesion, proliferation, and growth of a smooth muscle cell culture [121]. Similarly, years later, Moulick et al. [181] developed another platform that allows the adhesion, growth, and maturation of primary neuronal cells on POPC lipid bilayers modified with neuronal adhesion proteins (EA5-FC) to test ligand–receptor interactions between cells as they occur in neuronal synapses.

In an effort to comprehend biochemical processes occurring at membrane surfaces, the role of heparan sulfate proteoglycans (HSPGs) associated with cell membranes was investigated by Caseli and colleagues [202]. The study analyzes the Syndecan-4 ability in the processes of epidermal growth factor (EGF) recognition. The researchers selected Syndecan-4 as a case study due to its classification as a type I HSPG transmembrane glycoprotein, which is involved in a number of biological processes, including cell adhesion, proliferation, intercellular signaling, and tissue morphogenesis. In order to validate the hypothesis that EGF does not penetrate the DPPC monolayer, Langmuir isotherms were employed, while polarization-modulated infrared reflection absorption spectroscopy (PM-IRRAS) was used to provide chemical confirmation of EGF binding to the sulfate chain.

Considering that proteins are involved in biochemical signaling processes, which is fundamental in cell regulation and communication, a novel characterization technique has been implemented over the past two decades [219]. Total internal reflection ellipsometry (TIRE) enables the quantification of interactions between ligands or drugs and membrane proteins, such as G protein-coupled receptors (GPCRs), without the need for labeling, protein purification, or reconstitution of membrane proteins [168]. The investigation into the interactions with this integral protein is relevant due to the fact that GPCRs include receptors for a considerable number of ligands, including hormones, neurotransmitters, and inflammatory mediators. It controls a wide range of physiological functions and is associated with approximately 30 human diseases, including diabetes, obesity, cancer, hypothyroidism, and psychotic disorders [220]. In a study conducted by Kriechbaumer et al. [168], the interactions between the ligand chemokine (CXCL12α) and the receptor CXCR4 on Ishikawa endometrial adenocarcinoma cells were analyzed using TIRE. The inhibitory effect of the CXCR4-binding drug AMD3100 was also investigated.

In 2020, Krajewska et al. [207] conducted a study analyzing lipid–protein interactions. The researchers prepared OA:(α-LA): Ca^2+^ systems using Langmuir monolayers and LB films. Their objective was to evaluate the monolayer stability as a function of ions, pH, temperature, and density of molecular packing. The systems exhibited structural stability independent of conformational alterations in response to varying environmental conditions, which enabled them to ascertain that there is a strong ability of fatty acids and proteins to form antitumoricidial complexes at the interface.

#### 4.1.3. Enzymes

Enzymes have been studied for their potential applications in biotechnology as biosensing devices. In a study conducted in 2008 using colorimetric assays, the applicability of enzyme immobilization as a biosensor was demonstrated by immobilizing urease on a monolayer of DPPG, where the enzymatic activity of the system was demonstrated by detecting the presence of urea [171]. Two years later, Goto et al. immobilized catalase on DPPG to evaluate the decomposition of H_2_O_2_, as high concentrations of this compound in the cellular environment cause numerous neurodegenerative pathologies, cancer, or diabetes [170]. Similarly, Rodrigues et al. immobilized cellulase and ADH in DPPC to evaluate the biotechnological effects of these enzymes in the production, identification, and/or control of second-generation ethanol [164]. Although enzymes are at risk of denaturation when immobilized, Langmuir films provide a non-denaturing environment due to the amphiphilic nature of the lipids and enzymes [170,221]. In some cases, the enzymatic activity is even higher in LB films than in homogeneous solution [164,170], as evidenced in the work published by Goto et al., where the enzymatic activity of catalase in homogeneous solution was 87% of that obtained in the DPPG–catalase film [16].

In 2008, Schmidt et al. [197] demonstrated that horseradish peroxidase (HRP) exhibits enhanced activity when immobilized in Langmuir–Blodgett (LB) films. One year later [198], horseradish peroxidase (HRP) was immobilized with chitosan in alternating layers from an aqueous solution. The enzymatic activity with pyrogallol was successfully monitored by optical microscopy for a considerable period of time, thereby establishing this system as an efficient biosensor for HRP. In light of the fact that Langmuir films represent an effective approach for regulating catalytic activities at the molecular level [221], an antitumor agent, asparaginase, was immobilized in DPPC monolayers. The incorporation of the enzyme was confirmed through PM-IRRAS, and fluorescence spectroscopy demonstrated that this system is well-suited for asparagine sensing [201].

The above-described incorporations of enzymes in the model cell membranes and their molecular interactions with cell membrane components have been verified and monitored using techniques such as Quartz Crystal Microbalance (QCM) with dissipation factor and frequency variation, ellipsometry, PM-IRRAS, and neutron reflection. These techniques allow for the determination of the mass of biomolecules adsorbed on the film surface, the thickness and viscosity of the film after exposure of the biomolecules, and the mechanism of intermolecular interaction through frequency dissipation graphs. This approach, as demonstrated by Nielsen et al., involves monitoring enzyme interactions with the membrane to evaluate the effect on membrane structure, integrity, or enzyme activities [173].

### 4.2. Methodologies for the Incorporation of External and Cell Membrane Components onto the Lipid Planar Model Cell Membranes

Depending on the cell model to be prepared, different methodologies can be applied. Among the models implemented to characterize and study molecular interactions are monolayers, lipid bilayers, and supported bilayers.

#### 4.2.1. Monolayers

The Langmuir monolayer preparation method has been implemented by several authors for the incorporation of different molecules, including enzymes. In general, after the spreading of the lipid components and evaporating the solvent, molecular incorporation of proteins and enzymes is performed by injecting molecular solutions a few millimeters below the air–water interface.

The incorporation of proteins and enzymes into a model cell membrane at the air–water interface has been achieved using different strategies [199,200], including (i) co-spreading with lipids and sterols, (ii) injecting the protein beneath an already formed model cell membrane at the air–water interface, (iii) spreading lipids and sterols onto an aqueous subphase containing a water-soluble protein, and (iv) separately spreading a protein solution on the water interface, either before or after lipid spreading. Interestingly, some researchers have focused their attention on the study of Gibbs monolayers of proteins. Since most proteins are water-soluble, they do not form Langmuir films, but they tend to reside between the air–water interface and the inner subphase. This results in changes in the surface tension of water and promotes studies on the dynamics of the adsorption/desorption process at the interface, as well as the aggregation mechanism of such proteins related to certain diseases (e.g., Parkinson’s or Alzheimer’s) [222]. These proteins can also be transferred onto solid supports for biosensing and other applications [191,192,223]. Another interesting advantage of Langmuir films is that they can be used to study transmembrane proteins because these films, even if they only mimic half of the membrane, are located at the air–liquid interface and there are no constraints imposed by a solid substrate [202,224].

#### 4.2.2. Bilayers

**Figure 8:**
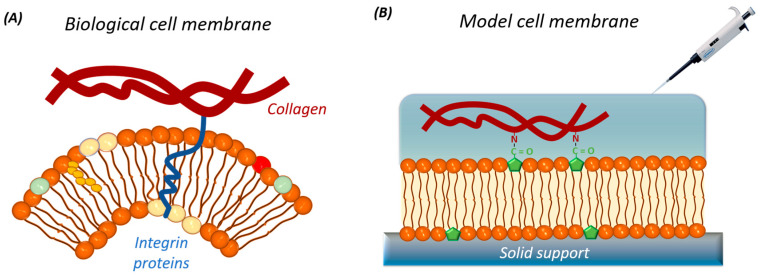
(**A**) Schematic representation of the natural binding of collagen fibers to the cell membrane via integrin proteins. (**B**) Chemical functionalization of a planar model cell membrane to incorporate collagen fibers, mimicking the natural cell membrane. Adapted with permission from [195]. Copyright 2010 American Chemical Society.

Among the methodologies implemented for protein incorporation, several procedures have been employed so far. Modifications of the vesicle rupture and fusion method have been used to prepare a lipid bilayer, onto which proteins are injected, adsorbed, and/or incubated [179,181,182,184,225]. In addition, the detergent-mediated reconstitution method in solution has also been employed [225,226]. This method involves the formation and reorganization of micelles by varying the concentration of the surfactant to incorporate integral or peripheral proteins, resulting in proteoliposomes (NOG-EA5) [181]. Subsequently, these proteoliposomes are deposited on the lipid bilayer, and through lipid hydration, sonication, and extrusion techniques, vesicles are prepared. Chemical functionalization of a component in the membrane has also been used as a strategy to induce the covalent bind of a protein. A good example is the paper by Huang et al., who constructed a biomimetic platform based on a phospholipid SLB functionalized with a carboxylic acid [195]. Subsequently, collagen molecules were introduced via amide linkages with the functionalized lipid (Figure 8).

Table 4, Table 5 and Table 6 gather illustrative examples of various membrane components, along with representative case studies employed to fabricate mono- or bilayers that mimic cell membranes. In these tables, it is possible to identify phosphatidylcholine, SM, and Chol as the most commonly incorporated molecules into model cell membranes. Phosphatidylcholine is often used in these model systems because this glycerophospholipid represents, on average, 50% of the phospholipid components in the eukaryotic cell membrane [30]. SM, a typical neuronal cell lipid, attracts the interest of the scientific community due to its relevance in medicine [227,228], and cholesterol represents 15–50% of the lipid components of both the eukaryotic plasma membrane and the membrane of cellular organelles [229,230].

## 5. Applications of Planar Model Cell Membranes

### 5.1. Model Cell Membranes as Platforms for Fundamental Knowledge Acquisition

As described above, the cell membrane plays a crucial role in the regulation, control, and communication of biological processes occurring in living organisms. One of the primary applications of cell membrane models is to gain fundamental insights that enhance our understanding of the nature of cellular membranes as well as for elucidating their interactions and functions and developing treatment systems to regulate cell membrane functions [28]. These models help elucidate how different components are organized, distributed, and interact within the membrane, thereby improving our comprehension of the physiological phenomena occurring at the cellular level. Essentially, these studies bridge the gap between the physicochemical principles at the interface and cellular mechanisms in both healthy and diseased cells. Throughout this review (see Table 3, Table 4, Table 5 and Table 6 for selected representative examples), we have explored how the interactions between various cellular membrane components have been investigated. In this context, Langmuir films serve as an excellent platform for studying mixed films containing two or more membrane components, providing a deeper understanding of molecular interactions. As demonstrated by numerous examples in this review, Langmuir monolayers have been employed to study the interactions of lipids with proteins, antimicrobial peptides, drugs, and other bioactive molecules, offering insights into how these molecules incorporate or adsorb onto membranes. Notable examples that remark the importance of these models include, but are not limited to, studies on pulmonary surfactants [231,232], tear films [233], model hydrophobic biological environments [234], and the aggregation of proteins leading to the dysfunction of physiological processes [222]. Black lipid membranes and supported membranes on solid surfaces have also been the focus of numerous fundamental studies aimed at replicating the structure and composition of cellular membranes on solid surfaces, facilitating the exploration of membrane functionality and chemical reactivity. These models have been extensively used for the immobilization of proteins, peptides, and other biomolecules, enabling the investigation of specific biochemical interactions [146]. They are essential for studying ionic transport across membranes, including ion channel modeling [98], to understand lipid raft formation and functionality [235], as well as for creating biocoatings for cardiovascular implants [19], and biomimetic platforms that eventually could allow the artificial recreation of organelles and cells [236,237,238]. Additionally, these models have been instrumental in electrophysiology studies, allowing researchers to explore the electrical properties of membranes, such as capacitance and resistance, to better understand conduction phenomena in cells. In recent years, advances in this field have extended to investigating various processes involving the cell membrane, including therapeutic treatment development, disease management strategies, vaccine formulation, biosensor creation for target substance detection, and the transport of active pharmaceutical compounds [41]. To this end, not only have biological components been incorporated into model cell membranes, but the modification of natural cell membrane components has emerged as a significant research interest. These modifications aim to improve the efficiency of incorporation, biocompatibility, transport, and delivery of drugs into the human body. Relevant examples include the covalent binding of polyethylene glycol (PEG) to membrane components to mitigate immune responses and to modify the surface properties of liposomes, thereby extending systemic circulation [189,239,240].

The knowledge gained from these fundamental studies has been made possible through the intelligent and synergistic combination of molecular nanoarchitectonic tools and a diverse array of characterization techniques, often used in tandem (correlation methodologies). Though a detailed analysis of these characterization techniques is beyond the scope of this review, readers may find a summary of the most commonly used techniques for characterizing planar model cell membranes, along with representative references for further information, in Table 7 and Table 8.

### 5.2. Alteration of Membrane Components to Promote Changes in the Performance of the Cell Membrane

Any substance that attempts to enter or leave the cell must pass through the membrane. Depending on the cellular needs, affinity, and external factors such as pH or osmotic pressure, the membrane will allow or not the passage of such compounds. It is important to note that the membrane is not composed solely of lipids but also contains proteins, enzymes, and exchange channels. These components not only add complexity to the membrane but also perform critical biological functions such as self-regulation, repair, and communication [300]. These functions have been extensively studied, especially through the use of model cell membranes. In this context, several studies have been conducted to evaluate the functionalization of the membrane with new proteins, enzymes, and other molecules. This approach aims to alter the local composition of the membrane, thereby promoting the anchorage of new substances, controlling and regulating cellular functions, and facilitating the propagation of messengers [301].

In particular, it has been shown that changes in the ratio of phospholipids can lead to the proliferation of cancer cells [301]. In addition, the interaction of fatty acids in surface modification has been linked to organ failure in cardiovascular diseases [302]. Alterations in the fluidity of mitochondrial protective bilayers affect ATP production and reactive oxygen species consumption, leading to lipid oxidation, ion channel opening, and mitochondrial damage, which may contribute to neurodegenerative diseases [303].

### 5.3. Transport and Encapsulation

The concept of treating diseases by specifically administering drugs to the target area has gained increasing attraction due to the ability to encapsulate active substances within artificial membranes. For this reason, liposomes and artificial vesicles made of membrane components have been proposed as vehicles to transport enzymes, genetic material such as DNA and RNA, drugs, or NPs. These systems make use of various biochemical mechanisms to control malignant cells or tissues [304].

Among the most important characteristics of membrane systems for substance encapsulation is their structural affinity with human cells, which helps them evade the immune system and results in prolonged half-lifes within the organism, leading to improved bioavailability [305]. On the other hand, their morphological characteristics allow them to encapsulate a wide variety of substances (both hydrophilic and hydrophobic) and provide selectivity for delivering the payload to specific sites. In addition, functionalization with polymeric materials can enhance their mechanical properties and their ability to respond to stimuli or interactions with other molecules. This enables precise control over the loading and unloading of active components [306].

Membrane systems facilitate the investigation and development of innovative therapeutic drugs by assessing factors such as toxicity, inhibition of ion exchange channels, changes in membrane permeability upon drug contact, partition coefficients to quantify the degree of encapsulation achieved, and other relevant parameters [5]. For this reason, model cell membranes offer a suitable platform for evaluating strategies to improve the efficacy of both encapsulation and treatment, including lipid/drug ratio, pH, salt concentration and medium polarity, degree of dissociation, loading, and interaction of potential drugs for the treatment of frequent and complex diseases [307].

A significant number of substances have been investigated due to their enhanced ability to control diseases when encapsulated. For example, *doxorubicin (DOX)*, a widely used cancer treatment drug, effectively inhibits growth and induces apoptosis (i.e., cancer cell death) when encapsulated and delivered directly to the affected area [308,309]. Moreover, other substances such as antifungals, antipsychotics, and antibiotics such as *azithromycin* have been studied due to their strong lipid-protein membrane interactions and their affinity for domains low in SM and Chol [5].

Furthermore, natural substances such as phytoalexins (resveratrol) have been investigated for their antiviral, antibacterial, and antifungal properties, as well as their beneficial effects on the cardiovascular system and their therapeutic effects against various types of cancer [27]. This study evaluates physicochemical parameters such as lipid packing, fluidity, and permeability of resveratrol at different concentrations in model membranes composed of POPC, SM, and Chol, contributing to the development of the optimal composition of liposomes.

Current investigations do not only use model cell membranes but also real membrane systems from cells, exosomes, etc., which are studied directly to further exploit their immunocompatibility, such as red blood cells. These membrane systems are treated through loading methods such as electroporation, osmosis, and incubations, among others, and thus evaluated for their effectiveness and applicability in clinical treatments [308]. Indeed, mesenchymal stem cells (MSCs) have been the subject of extensive investigation as potential nanocarriers due to their tumor-tropic properties and low immunogenicity, which suggests a high potential for use in diagnostic and therapeutic strategies [310,311,312]. For instance, MSCs were employed in numerous clinical trials conducted during the SARS-CoV-2 pandemic to develop vaccines based on DNA or mRNA encapsulated in lipid nanoparticles (LNPs) [313,314,315,316]. By mimicking cell membranes, COVID-19 *Pfizer/BioNTech and Moderna vaccines* use ionizable cationic lipids, PEG–lipids, DSPC, and Chol [189,190]. In addition, some clinical trials are incorporating nucleic acids, tumor antigens, neoantigens, and tumor-associated antigens loaded into liposomes as vaccines to treat infectious diseases such as rabies, Zika virus, tuberculosis, influenza, and cancer immunotherapy for glioblastoma, melanoma, breast, ovarian cancer, etc. [317,318,319,320,321,322,323].

### 5.4. Functionalization of Nanoparticles

Over recent years, it has become evident that functionalized NPs have the potential to make a substantial contribution to the field of biomedical research, particularly in areas such as bioimaging, biosensors, drug delivery, antimicrobial, cancer, inflammatory, and diabetes therapy, due to their stability, circulating half-life, required biodistribution, and passive or active targeting [324,325,326]. NPs can be classified into (i) polymeric NPs (Poly-Butylcyanoacrylate PBCA and poly lactic-co-glycolic acid PLGA), chitosan, lipid, solid lipid NPs, lipid nanocapsules (LNCs), carbon nanomaterials, lyotropic liquid crystalline nanoparticles (LLCNPs), etc.; and (ii) inorganic NPs that comprise silver, gold, selenium, silica, iron oxide, platinum, titanium dioxide, palladium, copper, metal sulfide, manganese oxide, zinc oxide, cerium oxide, magnetic NPs, among others [325,327,328].

In light of the shared attributes of mesenchymal stem cells (MSCs), neural stem cells (NSCs), and hematopoietic stem cells (HSCs) and the adaptable nature of NPs in cancerous tumor diagnosis and treatment, several researchers have attached NPs to steam cells as a strategy for targeted delivery with low cytotoxicity [329,330,331,332]. In view of the physiological obstacles presented by factors such as toxicity and the restricted capacity to encapsulate sufficient quantities of drugs with activated release, researchers continue to design and synthesize functionalized NPs that can overcome these challenges. These include mesoporous silica NPs, core–shell structures, yolk–shell/nanorattles, and Janus-structured particles, which are notable for their compatibility, high drug loading, and sustained drug release properties [312,329,333]. In addition, the development of coatings comprising gold and Fe_3_O_4_ NPs has enabled the creation of biomaterials exhibiting plasmonic, optical, acoustic, and magnetic properties, respectively [334,335]. These nanomaterials have potential applications in diagnosis and the integration of chemotherapy, photodynamic therapy (PDT), and photothermal therapy (PTT) treatments [312,334,336,337].

### 5.5. Biosensors

As the lipid membrane is considered an ideal platform for the study and interaction of molecules with enzymes, proteins, peptides, nucleic acids, nanomaterials, and polymers, among others, many investigations have constructed planar model cell membranes that respond to these interactions for their use as biosensors [233,234]. One of the principal challenges in biosensing is to preserve the functionality of the biomolecules used as transducer materials within the immobilized layers. To address this, Siqueira et al. employed two preparation methods that allow precise control of the molecular architecture within the film, namely the Layer-by-Layer and LB methods [338].

Biosensing platforms rely on the anchoring of target molecules to previously prepared ligands. This anchoring triggers a signal that not only detects but also quantifies the number of molecules present in the medium. To accomplish this, biomarkers—often contained within exosomes or vesicles—are released and detected upon anchoring, either spontaneously or through external stimuli, using conventional characterization techniques [339]. Various detection methods have been employed to identify these target molecules, including amperometric measurements, cyclic voltammetry, electrical impedance spectroscopy, colorimetric intensity, chemiluminescence, UV-vis absorption, and fluorescence spectroscopy, sum frequency generation (SFG) vibrational spectroscopy, QCM, Atomic Force Microscopy (AFM), and Surface Plasmon Resonance (SPR) [198,264,276,285,293,338,339,340,341,342,343,344,345]. Advances in biosensor technology have enabled the modification of cell membranes to produce biological detectors that can monitor ions such as Mg^2+^, Pb^2+^, and Zn^2+^ in real time. This is achieved by using genetically engineered membrane cells with specific DNA sequences designed to capture these ions on their surfaces [346,347].

## 6. Conclusions and Future Directions

Throughout this review paper, we have shown that researchers currently have access to a wide variety of relatively simple methodologies for modeling cell membranes. This is of great interest to enhance our understanding of the functional mechanisms of these membranes. Since the cell membrane acts as the barrier that separates the cell from the external environment, understanding how different agents—including natural compounds, drugs, ions, and other substances—interact with the membrane and what allows or prevents the passage of these agents into the cell is crucial in modern biology and medicine. This knowledge is not only essential for designing new drugs but also for developing innovative drug delivery systems that appropriately interact with the cell membrane. The ability to simulate a cell membrane in the laboratory opens up numerous applications, including the early detection of certain diseases through the design of biosensors. By considering this application, for which there are already promising early examples in the literature, future research should focus on improving the sensitivity, reproducibility, and scalability of these biosensors. This would enable their use in simple and cheap analytical procedures for the reliable and early detection of diseases, thereby preventing their progression and spread.

In addition, the use of membranes (both real and modeled) for encapsulating drugs or functionalizing nanoparticles (NPs) is a promising strategy to evade the immune system during drug delivery, thereby increasing bioavailability and organotropism. In the short term, efforts seem to be focused on synthesizing hybrid nanosystems that combine real and modeled cell membranes. This approach aims to harness the benefits of real systems (such as cellular recognition, organotropism, and low toxicity) while exploiting the advantages of modeled systems (such as control, reproducibility, and the ability to incorporate bespoke functional components). Such nanofabrication should prioritize environmentally friendly and biocompatible processes. Importantly, cells and extracellular vesicles may contain undesirable genetic information that could promote metastasis or other pathologies. Therefore, a significant challenge today is the removal of this material while preserving the relevant membrane components and structure to achieve the aforementioned advantages (low immunogenicity and high organotropism). In the medium to long term, it is anticipated that the necessary expertise will be developed for the production of entirely artificial systems (such as vesicles, liposomes, and exosomes) using mass production methods. These systems should ideally exhibit high biocompatibility, low immunogenicity, low toxicity, high stability under physiological pH and temperature conditions, good pharmacokinetics (long half-life), excellent biodistribution (capable of crossing not only the cell membrane but also the blood–brain barrier), targeted delivery, organotropism, and minimal side effects.

## Figures and Tables

**Table 7:** Techniques used in the characterization of Langmuir films as model cell membranes.

Characterization Technique	Principle	Information Provided	References
Surface pressure vs. area per molecule isotherms	Measurement of surface tension changes as molecules are compressed on the air–water interface	Measurement of surface tension changes as molecules are compressed on the air–water interface. Compression modulus. Excess thermodynamic properties in multicomponent films.	[145,241]
Surface potential vs. area per molecule isotherms	Measurement of the electrical potential difference across the air–water interface	Surface potential changes related to molecular orientation and interactions	[159,242,243]
Fluorescence Microscopy	Fluorescence detection at the air–water interface	Visualization of morphology, domain formation, and phase separation	[244,245]
Brewster Angle Microscopy (BAM)	Reflection of a p-polarized laser beam at the Brewster angle (~53° for water) on the air–water interface containing a monolayer	Real-time visualization of monolayer organization and molecular orientation	[246,247,248]
X-ray diffraction (XRD), grazing incidence X-ray diffraction (GIXD), X-ray specular reflectivity (XR), and neutron specular reflectivity	Diffraction of X-rays by ordered molecular structures at the air–water interface	Structural information, molecular packing, and phase behavior	[249,250,251,252,253,254]
Ellipsometry	Change of ellipsometry angles associated with reflection	Layer thickness and density	[255,256]
Reflection/Absorption of electronic (UV-vis and fluorescence) and vibrational spectroscopies (IR and Raman)	Measurement of electron transitions or vibrational modes of molecules at the air–water interface	Molecular composition, chemical interactions, formation of aggregates, and structural changes	[257,258,259,260,261,262]

**Table 8:** Characterization techniques commonly used for the characterization of supported lipid bilayers, black lipid membranes, and self-assembly monolayers.

Characterization Technique	Principle	Information Provided	References
Quartz crystal electrochemical microbalance, QCM with dissipation (QCM-D), and impedance-based QCM (QCM-Z)	Oscillation frequencies of a quartz crystal that depends on the amount of material deposited on the surface	Real-time adsorbed mass and energy dissipation	[263,264,265]
Electrochemisty: cyclic voltammetry (CV) and electrochemical impedance spectroscopy (EIC)	Resistance of an electrochemical system to the flow of electrical current	Real time: Interfacial properties related to bio-recognition	[266,267]
Neutron reflectometry (NR)	Measures the intensity of neutrons reflected from a surface to determine the structure and composition of thin films and interfaces	Structure, composition, and interactions	[268,269,270]
X-ray reflectometry (XRR)	Measures the intensity of X-rays reflected from a surface to assess the thickness, density, and roughness of thin films and interfaces	Structural properties and phase transitions	[271,272]
Ellipsometry	Change of ellipsometry angles associated with reflection	Interfacial mass and layer thickness and density	[135,273]
X-ray fluorescence	Monochromatic synchrotron X-ray beam irradiating the sample causing the atoms in the sample to emit secondary (or fluorescent) X-rays	Analyze the composition for detecting the presence and distribution of metal ions or other elements	[274]
Surface plasmon resonance (SPR)	Excitation of molecules with lasers and monitoring of the emitted spectrum	Real-time monitoring of morphology and physicochemical properties	[275,276,277]
Total internal reflection ellipsometry (TIRE)	Combination of ellipsometry and SPR to detect changes in the polarization of reflected light	Adsorption/desorption Quantify membrane receptor interactions at the surface	[168]
Nanoplasmonic Sensor (NPS)	Localized surface plasmon resonance induced by refractive index changes around the model cell membrane induced by molecular binding events	Mass, thickness, and conformation of adsorbates, interaction kinetics, and binding avidity	[278,279,280,281]
Surface plasmon fluorescence spectroscopy	Intensity of electromagnetic field and excitation of surface plasmons	Topography and physicochemical properties	[275,282]
Sum Frequency Generation Vibrational Spectroscopy (SFG)	Nonlinear optical technique based on the generation of sum frequency from two incident light beams	Information on interfacial peptide and protein structure (e.g., conformation and orientation) and interactions between peptides and proteins with lipid layers	[283,284,285,286]
Attenuated-total reflectance Infrared spectroscopy (ATR-FTIR); surface enhanced infrared absorption spectroscopy (SEIRAS); and polarization modulated infrared reflection absorption spectroscopy (PM-IRRAS)	Infrared beam intensity or absorption of polarized light at specific angles/incident light, which is characteristic of the functional groups and order state of the material	Chemical composition, molecular orientation, lipid phase, and protein interactions	[57,137,208,287]
Fluorescence microscopy	Fluorescence detection (UV or blue light)	Interfacial morphology and phase structure	[288,289]
FRAP: fluorescence recovery after photobleaching	Fluorescence recovery in a photobleached area	Protein diffusion rates based on the fluorescence recovery rate within a photobleached region in the model membrane	[290,291]
Atomic force microscopy (AFM)	Interaction force between surface and detection tip	Surface topography, layer thickness, mechanical properties, interactions on a surface, phase separation, nanomechanical properties	[292,293,294,295,296,297]
Solid-state Nuclear magnetic resonance (NMR)	Radiation absorption in atomic nuclei under a magnetic field	Composition, lipid–protein interactions, and structure 3D of proteins embedded in a lipid bilayer	[298,299]

## Data Availability

No new data were created or analyzed in this study. Data sharing is not applicable to this article.
